# Effects of climate‐change scenarios on the distribution patterns of *Castanea henryi*

**DOI:** 10.1002/ece3.9597

**Published:** 2022-12-08

**Authors:** Chunping Xie, Erlin Tian, Chi Yung Jim, Dawei Liu, Zhaokai Hu

**Affiliations:** ^1^ College of Science Qiongtai Normal University Haikou China; ^2^ Department of Social Sciences Education University of Hong Kong Tai Po Hong Kong China; ^3^ Nanjing Forest Police College Nanjing China; ^4^ Guangdong Ocean University Zhanjiang China

**Keywords:** bioclimatic factors, *Castanea henryi*, climate‐change scenario, habitat suitability, MaxEnt model, species distribution models (SDMs)

## Abstract

*Castanea henryi*, with edible nuts and timber value, is a key tree species playing essential roles in China's subtropical forest ecosystems. However, natural and human perturbations have nearly depleted its wild populations. The study identified the dominant environmental variables enabling and limiting its distribution and predicted its suitable habitats and distribution. The 212 occurrence records covering the whole distribution range of *C. henryi* in China and nine main bioclimatic variables were selected for detailed analysis. We applied the maximum entropy model (MaxEnt) and QGIS to predict potentially suitable habitats under the current and four future climate‐change scenarios. The limiting factors for distribution were accessed by Jackknife, percent contribution, and permutation importance. We found that the current distribution areas were concentrated in the typical subtropical zone, mainly Central and South China provinces. The modeling results indicated temperature as the critical determinant of distribution patterns, including mean temperature of the coldest quarter, isothermality, and mean diurnal range. Winter low temperature imposed an effective constraint on its spread. Moisture served as a secondary factor in species distribution, involving precipitation seasonality and annual precipitation. Under future climate‐change scenarios, excellent habitats would expand and shift northwards, whereas range contraction would occur on the southern edge. Extreme climate change could bring notable range shrinkage. This study provided a basis for protecting the species' germplasm resources. The findings could guide the management, cultivation, and conservation of *C. henryi*, assisted by a proposed three‐domain operation framework: preservation areas, loss areas, and new areas, each to be implemented using tailor‐made strategies.

## Graphical abstract

Determined present suitable habitat conditions for *Castanea henryi* in China; Modeled the species' potential distribution under future climate‐change scenarios; Potential suitable habitats shifted northwards under moderate climate change.

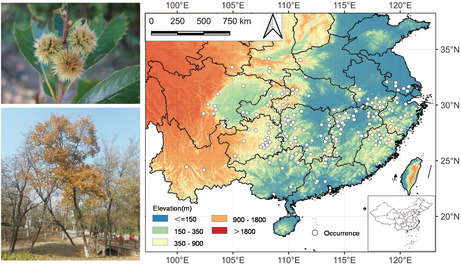

## 1 INTRODUCTION

Climate is one of the most critical factors molding the geographical distribution of species and the reproduction and growth of plants (Akyol et al., 2020). Understanding the species' geographical distribution and climatic correlation can grasp the macro‐scale pattern of their threatened status, endemism, diversity, evolution, and development and offer hints to predict future distribution (Wang et al., 2018). Global warming is causing the fragmentation of suitable habitats for plants. Some species have been driven to the verge of extinction, and some are triggered to migrate to areas with suitable climate conditions (Akyol et al., 2020; Huang et al., 2020). Therefore, studying the potential distribution of species and the impact of future climate on plant distribution is crucial for timely planning and strategy formulation in species diversity conservation (Li, 2019).

In recent years, some novel GIS‐based methods have played an important role in biogeography research (Booth et al., 2014; Fois et al., 2018). They have helped to develop models for predicting species distribution and simulating and visualizing species dispersal and migration pathways (Sarıkaya & Orucu, 2019; Tulowiecki, 2020). In general, these species distribution models (SDMs) integrate multiple environmental variables affecting the distribution of a species and match its actual distribution points with corresponding environmental variables to assess the species' niche requirements (Booth et al., 2014; Fourcade et al., 2018). The habitat preference is reflected in probability values. Finally, the suitable distribution range of the target species is obtained and depicted on a map (Akyol et al., 2020; Çoban et al., 2020; Huang et al., 2020). With the rapid development of computer and geographic information technologies, many models have been developed to evaluate the correlation between species and environmental variables based on different principles and algorithms. The more commonly adopted SDMs include GARP, BIOCLIM, DOMAIN, CLIMEX, and MaxEnt (Booth et al., 2014; Carpenter et al., 1993; Sarquis et al., 2018).

MaxEnt (maximum entropy) is the most widely used SDM with a high prediction accuracy (Abolmaali et al., 2018; Xie et al., 2021). The model describes a probability distribution where each grid cell has predicted suitable conditions for a species using a collection of environmental (e.g., climatic) grids and species georeferenced occurrence locations (Steven J. Phillips & Dudík, 2008). The investigation generates the anticipated probability of presence or forecasts local abundance under specific assumptions about the input data and biological sampling operations producing the occurrence records (Fourcade et al., 2018). MaxEnt has been widely used in predicting suitable areas for species growth. In Europe, future climate change may shift tree species range eastward, accompanied by range shrinkage in southern Europe (Puchałka et al., 2021). Other tree species range may shift northward in Europe due to increased climatic seasonality, whereas the southern edge may experience range contraction (Paź‐Dyderska et al., 2021). Different populations of the same plant species located at the northern and southern edges of the range could often express opposite reactions to a warming climate (Matías et al., 2014). However, such physiological resilience leading to buffering against range shrinkage may not last with increasing warming (Doak & Morris, 2010). MaxEnt has improved our understanding of the relationship between environmental factors and species growth, occurrence, and spatial changes. Therefore, MaxEnt results can provide hints to enhance the conservation and management of species in a specific region (Manish & Pandit, 2019).

*Castanea* Mill. is a relatively small genus in the Fagaceae family, with about 12 species mainly distributed in the temperate and subtropical regions of the northern hemisphere (Jones, 1986). Compared with other Fagaceae genera, *Castanea* has received limited attention in biogeography research (Deng et al., 2018; Manos & Stanford, 2001). The potential reintroduction of Chestnut to the U.S.A. was assisted by research on its geographic distribution (Dalgleish et al., 2015; Tulowiecki, 2020). SDM analysis permitted the identification of potential restoration sites based on two critical predictors: preference for acidic soil pH and relatively steep slope (Tulowiecki, 2020). Anatolian Chestnut's (*C. sativa*) potential and future distribution areas in Turkey were predicted to shrink significantly in response to climate change (Sarıkaya & Orucu, 2019). However, the geographic study of *C. henryi* has received little attention (Liu & Fang, 2000).

Only three species of *Castanea* are native to China, including *C. mollissima*, *C. seguinii*, and *C. henryi* (Skan). *C. mollissima*, with the longest cultivation history, has been studied intensively (Zhang, Wang, et al., 2022). *C. seguinii* has extremely small nuts and is planted for its edible nuts sometimes also, however not as widely as *C. mollissima* (Zulfiqar et al., 2019); therefore, studies on it are relatively rare. *C. henryi* began to be cultivated in northern Fujian Province (China) 400 years ago, yielding a well‐known edible dried nut in southern China (Gong & Chen, 1997). At present, research on *C. henryi* focuses on germplasm resources, genetic diversity, cultivation techniques, pest control, fruit storage, and processing (Ma et al., 2013). However, compared with *C. mollissima*, the research on *C. henryi* calls for a substantial increase in depth.

*Castanea henryi* has significant economic and ecological values and is essential in maintaining the function of subtropical forest ecosystems in China (Ma et al., 2013). However, a combination of climate change and human disturbance has negatively impacted its growth and distribution. Human activities have continuously degraded the forest habitats in China to curtail their survival and reproduction (Liang et al., 2018). In addition, climate change has altered its physiological and ecological traits to modify its distribution pattern (Fahad et al., 2021). Field surveys confirmed the rare occurrence of large trees of *C. henryi*, and the remaining young plants were relatively scarce (Li, Sun, et al., 2018). It tends to be replaced by other broad‐leaved trees (Li, Sun, et al., 2018; Liang et al., 2018).

We need to deepen our understanding of the factors that promote and limit the natural growth and dispersion of *C. henryi*. The findings could contribute to its conservation, selection of suitable cultivation sites, and sustainable utilization of the timber tree. Past studies had shown that climate change posed a serious socioeconomic and ecological risk to Anatolian Chestnut in Portugal; a gradual decrease in Chestnut Suitability Index (CSI) was observed for future climates, especially for RCP8.5 and over the long term (Freitas et al., 2022). Therefore, we assume that a similar situation will also occur in China. Applying the MaxEnt modeling method, the study objectives are to (1) identify and assess the significant climatic factors influencing the current distribution of *C. henryi*; (2) predict the best regions for future growth of *C. henryi* based on its current distribution data; and (3) forecast the future climatic trends and find the best regions for *C. henryi* growth under various climate‐change scenarios.

## 2 MATERIALS AND METHODS

### 2.1 Collecting species occurrence data

**FIGURE 1:**
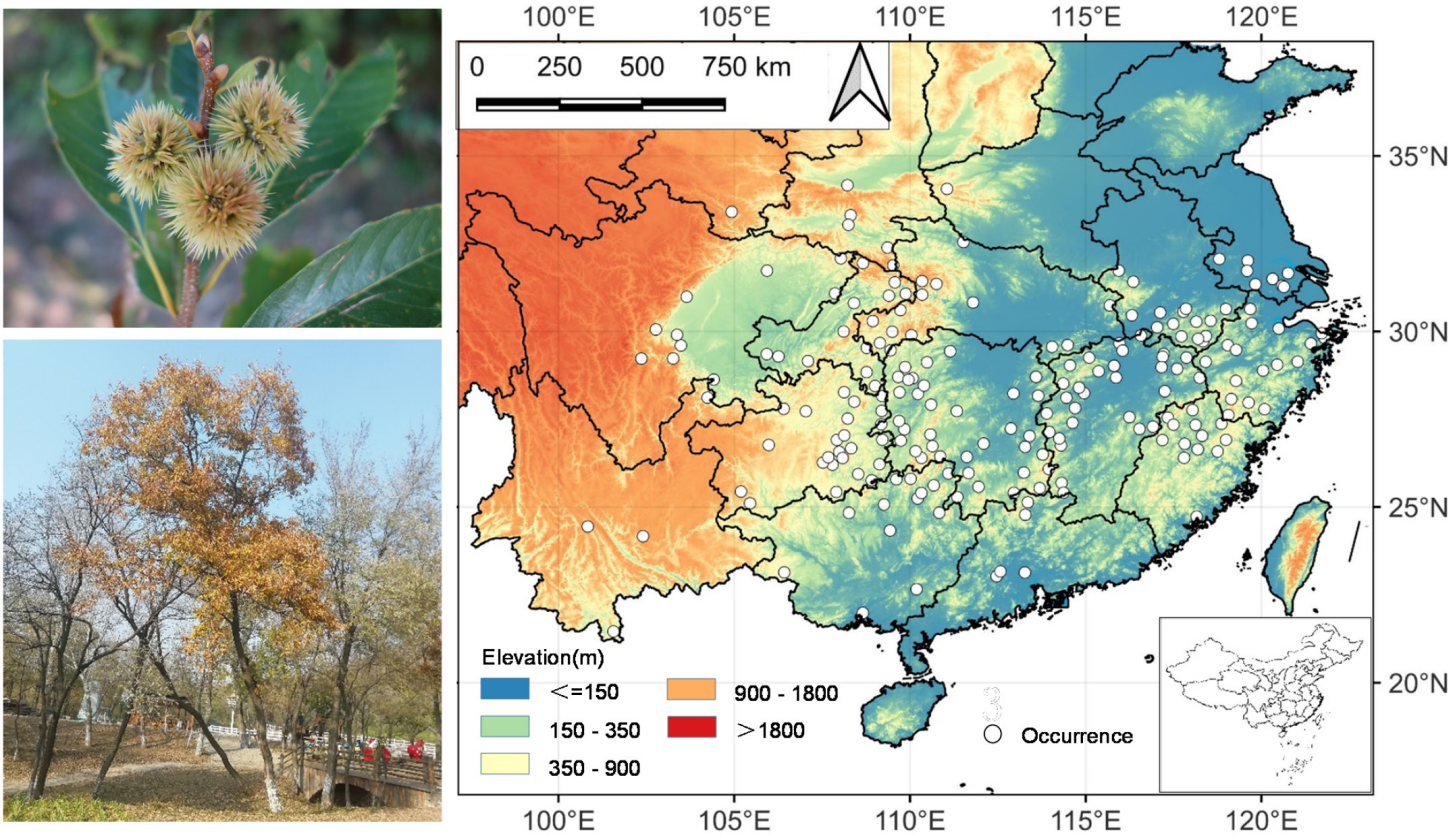
Locations of 212 selected occurrence records (shown by white dots) of *Castanea henryi* in China and images of a sample tree and flowers.

The geographical distribution data of *C. henryi* were obtained from field investigations, local flora, and database platforms. The databases include the Global Biodiversity Information Facility (GBIF, www.gbif.org), National Specimen Information Infrastructure (NSII, http://www.nsii.org.cn/), and Chinese Virtual Herbarium (CVH, http://www.cvh.ac.cn/). Some data were collected from CNKI (China National Knowledge Infrastructure) and Scopus. A total of 1551 initial records were obtained. They were screened to ascertain whether they were natural or cultivated and to remove the repeated, incomplete, and inaccurate records. To avoid errors caused by the high occurrence density, a sample cell size of 2.5′ grid was adopted. Using the QGIS software (www.gisagmaps.com/qgis‐download/), one record closest to each grid's center was kept, and the remaining points were deleted. Finally, 212 high‐quality and reliable records were kept (Figure 1). The records' geographical coordinates were acquired from Google Earth and saved in .csv format. The vector map of China was downloaded from the National Geomatics Center of China (NGCC, http://ngcc.sbsm.gov.cn/).

### 2.2 Selecting environmental variables

Bioclimatic variables for current and future climate scenarios were extracted from WorldClim (http://www.worldclim.org/). More biologically significant variables were derived from bioclimatic factors related to monthly temperature and rainfall records. They were frequently employed in simulating species distribution and associated ecological modeling. The bioclimatic variables represent seasonality, extreme or limiting environmental elements, annual trends (e.g., mean annual temperature, and annual precipitation), and annual range in temperature and precipitation (e.g., temperature of the coldest and warmest month and precipitation of the wet and dry quarters). The 19 specific bioclimatic variables chosen in this study (Bio1‐Bio19) are listed in Table 1.

**TABLE 1:** Nineteen environmental variables used in predicting the geographical distribution of *C. henryi*.

Bioclimatic variable	Code	Unit	Bioclimatic variable	Code	Unit
Annual mean temperature	Bio1	°C	Mean temperature of the coldest quarter	Bio11	°C
Mean diurnal range	Bio2	°C	Annual precipitation	Bio12	mm
Isothermality (Bio2/Bio7) (×100)	Bio3	Index	Precipitation of the wettest month	Bio13	mm
Temperature seasonality	Bio4	Index	Precipitation of the driest month	Bio14	mm
Max temperature of the warmest month	Bio5	°C	Precipitation seasonality	Bio15	Index
Min temperature of the coldest month	Bio6	°C	Precipitation of the wettest quarter	Bio16	mm
Temperature annual range	Bio7	°C	Precipitation of the driest quarter	Bio17	mm
Mean temperature of the wettest quarter	Bio8	°C	Precipitation of the warmest quarter	Bio18	mm
Mean temperature of the driest quarter	Bio9	°C	Precipitation of the coldest quarter	Bio19	mm
Mean temperature of the warmest quarter	Bio10	°C			

The current climate is the average data from 1970 to 2000 from the WorldClim version 2.1 (Fick & Hijmans, 2017). BCC‐CSM2‐MR (Beijing Climate Center Climate System Model; Wu et al., 2019) uses the SSP 1–2.6, SSP 2–4.5, SSP 3–7.0, and SSP 5–8.5 scenarios of the shared socioeconomic pathways (SSPs) adopted by the IPCC 6th Assessment, with a spatial resolution of 5 × 5 km for bioclimatic data in WorldClim v 2.1 (Wu et al., 2021). SSP scenarios are based on current national and regional realities and development plan to obtain specific socioeconomic development scenarios. They are upgraded versions of representative concentration pathways (RCPs) that reflect the link between socioeconomic development patterns and climate‐change risks (Kriegler et al., 2012). The BCC‐CSM2‐MR model was derived from the Coupled Model Intercomparison Project 6 (CMIP6), which provided effective and reasonable simulations of extreme temperature indices and their trends in China and global land areas (Wu et al., 2019, 2021). The chosen SSP‐RCPs denote combinations of (a) low societal vulnerability and low emission levels (RCP2.6, radiative forcings 2.6 W/m^2^ in 2100 and the same abbreviation protocol for the RCPs below); (b) intermediate societal vulnerability and intermediate emission levels (RCP4.5); (c) relatively high societal vulnerability and medium to high forcing levels (RCP7.0); and (d) RCP8.5 for higher emissions imposing high mitigation but low adaptation challenges (Mondal et al., 2021; O'Neill et al., 2016; Yang et al., 2022). Future climate projections were selected for 2041–2060 (2050s) and 2061–2080 (2070s).

Relatively strong correlations between the 19 bioclimatic variables could affect the simulation results. The variables were screened to tackle this multicollinearity problem. Spearman correlation analysis and variance inflation factors (VIF) were used to select and keep variables with correlation coefficients less than 0.8 (Abdelaal et al., 2019). Finally, nine bioclimatic variables were retained: Bio2, Bio3, Bio8, Bio10, Bio11, Bio12, Bio15, Bio17, and Bio18.

### 2.3 Model construction

The ENMeval package in R 4.0.2 (R Core Team, 2020) was used to optimize the MaxEnt model (Muscarella et al., 2014). The block method was used to divide the 212 *C. henryi* records into four parts, three for training and one for testing. We set the regularization multiplier (RM) to 0.5–4.0 with a 0.5 interval to yield 8 RM. The MaxEnt model provided five feature options: linear (L), quadratic (Q), hinge (H), product (P) and threshold (T) (Nottingham & Pelletier, 2021). We selected six feature combinations: L, LQ, H, LQH, LQHP, and LQHPT. The ENMeval package was used to test the 48 parameter combinations obtained by multiplying 8 RM by 6 feature combinations. The Akaike information criterion correction (AICc) was used to evaluate the fit and complexity of the model (Yan et al., 2021). The difference between training and testing AUC (area under the curve receiver operating characteristic) and the 10% training omission rate was used to evaluate the degree of overfitting of the model. The parameter combination had the minimum delta. The AICc value had been chosen as our optimal parameter to build the model (Steven J Phillips et al., 2017).

In this study, the model's accuracy was assessed by AUC, with a larger value indicating a higher accuracy of the prediction results (Abdelaal et al., 2019). AUC of 0.5–0.6, 0.6–0.7, 0.7–0.8, 0.8–0.9, and >0.9 indicated failure, poor, fair, good, and excellent categories for the predicted results, respectively (Abolmaali et al., 2018; Xie et al., 2021). The survival probability of *C. henryi*, indicated by the probability *p*, was reclassified into four categories of habitat suitability categories: poor (*p* < .25), fair (0.25–0.50), good (0.50–0.75), and excellent (>0.75). The raster tool in QGIS was used to calculate the suitable area. The raster tool in QGIS was used to calculate the suitability areas (QGIS, 2021).

## 3 RESULTS

### 3.1 Geographical distribution of *C. henryi* in China

The distribution range of *C. henryi* in China is roughly between 100.83° E − 121.80° E and 21.46° N–34.16° N (Figure 1). The southernmost point of the natural distribution was in the tropical zone (101.56° E，21.46° N), the northernmost point was close to the southern temperate zone (108.22° E, 34.16° N), the easternmost point was in the central subtropical zone (121.80° E, 29.81° N), and the westernmost point was in the tropical zone also (100.83° E, 24.45° N). Generally, the natural areas were mainly distributed in the typical subtropical region in China. The distribution pattern of *C. henryi* in China could be divided into four zones: Eastern, Central, Southwest, and Southern China. Eastern China included Fujian, Zhejiang, Anhui, Jiangsu, and Shanghai. Central China included Jiangxi, eastern Hunan, and the northern part of Guangdong. Southwest China included northern Hunan, southeastern Guizhou, Chongqing, northern Hubei, the Sichuan basin, Yunnan, and parts of Shaanxi, Gansu and Henan. South China is dominated by the southern part of Guangdong and Guangxi. Although belonging to the subtropical region, Taiwan has no occurrence record.

The altitudinal distribution of *C. henryi* was approximately 0–2000 m, with the highest occurrence record at Shennongjia in Hubei (ca. 1900 m) and the lowest at Suzhou in Jiangsu (ca. 5 m). Some 166 records (78.30%) had low‐altitude distribution (0–600 m), 39 records (18.40%) medium (600–1200 m), and 7 records (3.30%) high (>1200 m). A few occurrence records with a high altitude occurred in the southwestern zone, while most low‐altitude records occurred in the eastern zone. This altitudinal pattern coincided with the macro‐scale landform pattern of China.

### 3.2 Key enabling and limiting climatic factors

**FIGURE 2:**
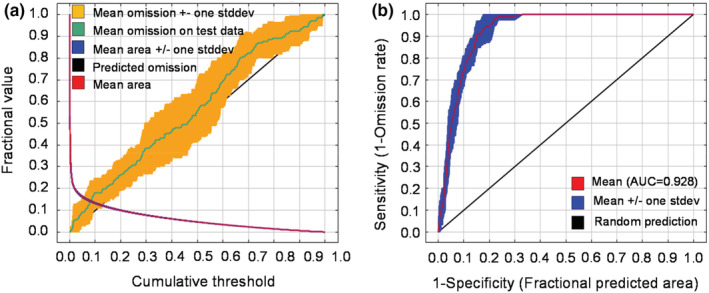
Measuring the accuracy of the generated MaxEnt models to predict the influence of environmental factors on *C. henryi* distribution: (a) omission rates versus predicted area; and (b) the area under the curve (AUC) of the receiver operating characteristics (ROC) curve.

**FIGURE 3:**
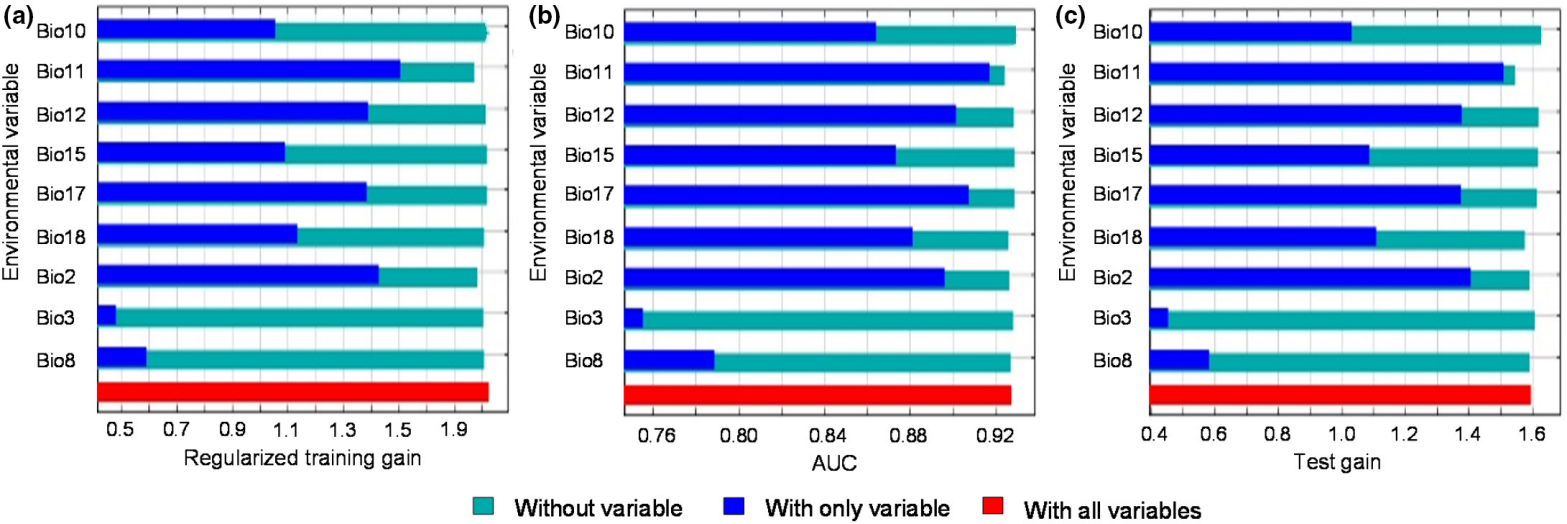
Jackknife plot of training gain for *C. henryi* indicating the differential influence of the selected environmental variables: (a) the environmental variable with highest gain when used in isolation is Bio11, which therefore appears to have the most information that is not present in the other variables; (b) the same jackknife test is according to AUC on test data.; (c) the same jackknife test using test gain.

The AUC value for the MaxEnt was 0.928 ± 0.014 (Figure 2). Thus, the model was a good predictor of the distribution area of *C. henryi*. The results (Table 2; Figure 3) indicated that mean diurnal range (Bio2), mean temperature of the coldest quarter (Bio11), precipitation seasonality (Bio15), isothermality (Bio3), and annual precipitation (Bio12) had a strong contribution to predicting the distribution. The mean temperature of the warmest quarter (Bio10) and mean temperature of the wettest quarter (Bio8) had a moderate influence on the distribution. Precipitation of the warmest quarter (Bio18) and precipitation of the driest quarter (Bio17) had the least effect on predicting the distribution (Table 2).

**TABLE 2:** Relative contributions and permutation importance of the nine environmental variables included in the MaxEnt model.

Environmental variable	Unit	Relative contribution (%)	Permutation importance
Mean diurnal range (Bio2)	°C	48.5	6.9
Mean temperature of the coldest quarter (Bio11)	°C	26.5	49.3
Precipitation seasonality (Bio15)	%	7.6	3.8
Isothermality (Bio3)	%	6.6	6.2
Annual precipitation (Bio12)	mm	4.8	12.8
Mean temperature of the warmest quarter (Bio10)	°C	2.7	6.1
Mean temperature of the wettest quarter (Bio8)	°C	1.5	3.5
Precipitation of the warmest quarter (Bio18)	mm	1.1	9.0
Precipitation of the driest quarter (Bio17)	mm	0.7	2.4

Bio2, Bio11, and Bio3 could be subsumed under temperature factors with a considerable cumulative contribution of 81.6%. Meanwhile, Bio15, Bio12, and Bio18 could be subsumed under moisture factors with a cumulative contribution of 13.5%. Therefore, the temperature had a notably greater influence on distribution than moisture. In addition, the top three bioclimatic variables in terms of permutation importance according to Jackknife were Bio11 (49.3%), Bio12 (12.8%), and Bio18 (9.0%) (Table 2). The results for both percent contribution and permutation importance suggested that temperature played a greater role than moisture, with Bio11 furnishing a greater weight than the other two bioclimatic variables. Meanwhile, the descriptive statistics of the main bioclimatic parameters in the distribution areas are also displayed in Table 3.

**TABLE 3:** Descriptive statistics of the main bioclimatic parameters in the distribution areas of *C. henryi* in China.

Bioclimatic variable	Mean	Standard deviation	Min.	Max.	95% confidence interval	Coefficient of variation %
Bio2	8.21	0.77	5.96	11.89	8.10–8.31	9.36
Bio3	27.66	3.84	22.65	55.05	27.14–28.18	13.87
Bio8	23.33	2.20	16.57	28.50	23.03–23.62	9.43
Bio10	26.63	1.61	17.30	28.97	26.41–26.84	6.03
Bio11	6.99	2.59	−1.77	17.80	6.64–7.34	37.02
Bio12	1381.29	266.07	541.00	2007.00	1345.27–1417.32	19.26
Bio15	60.37	11.24	42.92	102.56	58.85–61.89	18.62
Bio17	126.82	48.29	9.00	200.00	120.28–133.35	38.08
Bio18	529.84	113.24	193.00	1126.00	514.51–545.18	21.37

**FIGURE 4:**
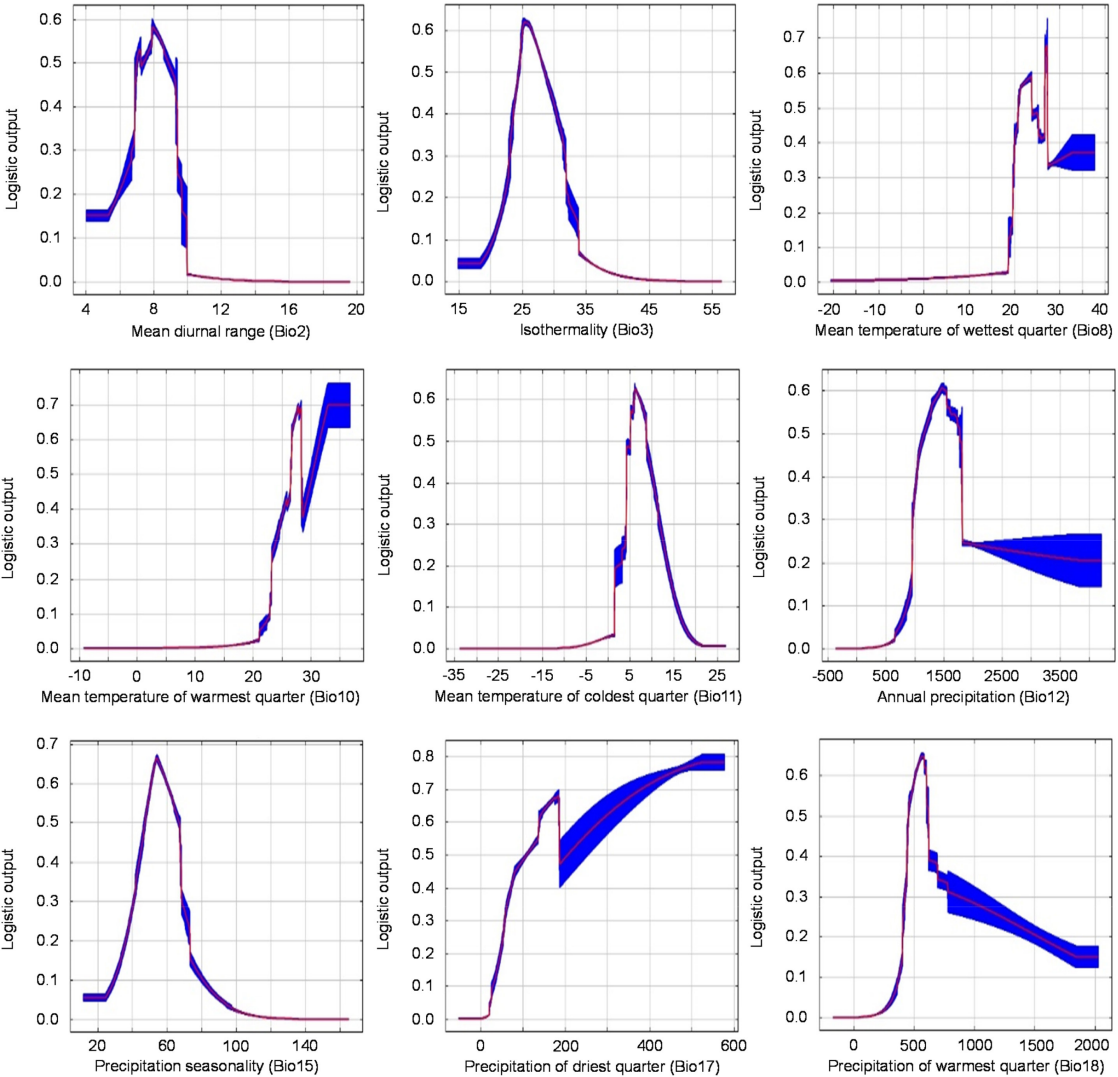
Response curves of nine environmental predictor variables used in the MaxEnt model for *C. henryi*. The curves show the mean response of the 10 replicate MaxEnt runs (red) and the mean +/− one standard deviation (blue).

The response curves in Figure 4 show how each environmental variable affects model predictions. The x‐axis indicates the value of an individual environmental variable, and the y‐axis is the projected likelihood of favorable conditions as determined by the logistic output. Variables displaying an upward trend show a positive association; those with a downward trend show a negative link. The magnitude of changes shows the strength of the relationship (G. Li et al., 2014). The changes in the response curve could provide helpful information on the environmental thresholds required for optimal growth (Akyol et al., 2020). It is generally accepted that when the distribution probability is >0.50, the corresponding environmental factor value is suitable for the species (Xie et al., 2021).

Among the temperature variables, the appropriate distributions for Bio2, Bio3, Bio8, Bio10, and Bio11 were about 6.5–9.0°C, 24–27%, 21–24°C, 25–27°C, and 5–9°C, respectively. Especially for Bio11, growth was not favored by excessively high or low average temperatures in winter. Among the moisture variables, the appropriate distribution ranges for Bio12, Bio15, Bio17, and Bio18 were about 1200–1700 mm, 48–68%, >100 mm, and 480–700 mm, respectively.

### 3.3 Potential distribution range under current climate

**FIGURE 5:**
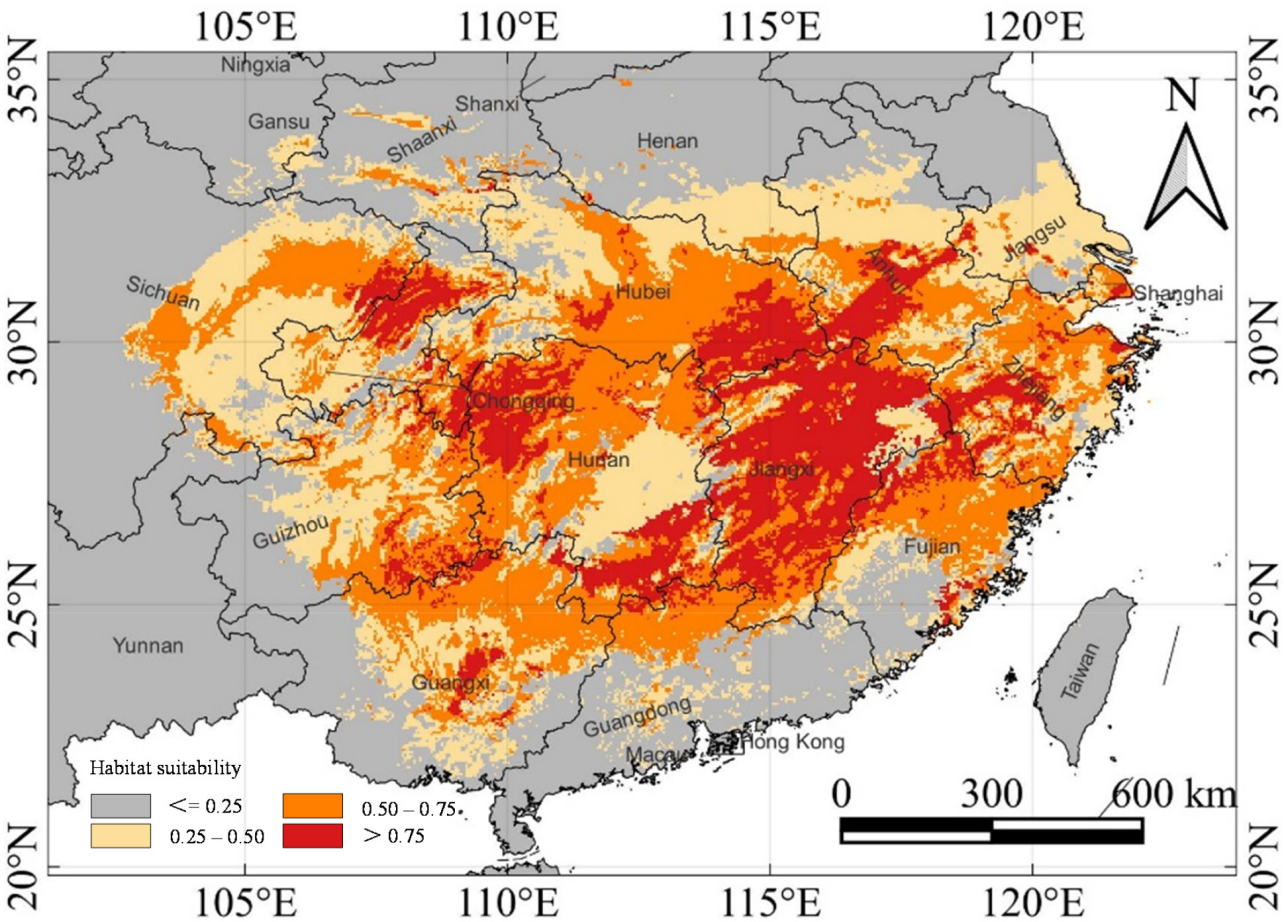
Potential suitability areas of *C. henryi* under the current climate scenario in China (1970–2000), divided into four categories based on the calculated habitat suitability index. Red color denotes excellent suitability habitat, orange good, yellow fair, and gray poor.

The area and distribution pattern of each habitat suitability category under the current climate were evaluated according to the habitat classification criteria (Table 4; Figure 5). The land areas for excellent, good, fair, and poor suitability were 26.98 × 10^4^ km^2^, 53.35 × 10^4^ km^2^, 54.36 × 10^4^ km^2^, and 825.32 × 10^4^ km^2^, respectively. The occurrence of poor suitability was predominant. The suitability area covered 134.69 × 10^4^ km^2^ or nearly 14% of China's total land area (Table 4). Jiangxi Province had the largest excellent‐habitat area under the current climate scenario (9.55 × 10^4^ km^2^), accounting for 35.4% of the excellent‐habitat area of China (Table 5).

**TABLE 4:** Predicting the habitat suitability areas of *C. henryi* under the current climate and four future climate‐change scenarios in 2050s and 2070s.

Climate scenario		Suitable habitat category							
		Poor (10^4^km^2^)	Ratio (%)^a^	Fair (10^4^km^2^)	Ratio (%)^a^	Good (10^4^km^2^)	Ratio (%)^a^	Excellent (10^4^km^2^)	Ratio (%)^a^
	Current	825.32	‐	54.36	‐	53.35	‐	26.98	‐
RCP2.6	2050	834.69	1.13	47.56	−12.52	53.34	−0.01	25.38	−5.94
	2070	838.66	1.62	46.46	−14.53	48.80	−8.53	27.04	0.21
RCP4.5	2050	842.22	2.05	48.59	−10.62	39.89	−25.23	30.26	12.17
	2070	835.23	1.20	50.31	−7.46	46.93	−12.04	28.49	5.61
RCP7.0	2050	836.39	1.34	49.38	−9.16	41.64	−21.95	33.55	24.33
	2070	838.40	1.58	44.29	−18.53	49.80	−6.65	28.47	5.53
RCP8.5	2050	835.14	1.19	51.32	−5.59	48.46	−9.17	26.05	−3.46
	2070	835.51	1.23	45.60	−16.12	51.09	−4.25	28.77	6.63

*Note*: The habitats have been divided into four suitability categories (10^4^ km^2^).

^a^ The ratio (%) of the habitat suitability areas of *C. henryi* to the current distribution under different climate scenarios.

**TABLE 5:** Predicted excellent suitable areas for *C. henryi* under the current and future climate scenarios in various provinces or autonomous regions of China (10^4^km^2^).

Location	Current	Future							
		RCP2.6 (2050s)	RCP2.6 (2070s)	RCP4.5 (2050s)	RCP4.5 (2070s)	RCP7.0 (2050s)	RCP7.0 (2070s)	RCP8.5 (2050s)	RCP8.5 (2070s)
Jiangxi	9.55	7.74	8.49	10.41	8.64	9.75	9.11	7.56	8.27
Hunan	4.85	5.36	5.32	5.80	3.90	5.76	5.83	4.90	5.06
Hubei	2.90	4.15	3.26	3.01	4.24	6.01	3.53	2.83	3.05
Anhui	1.92	1.34	2.62	2.09	1.42	3.00	1.81	1.95	2.83
Chongqing	1.77	0.69	0.78	1.23	1.26	1.05	1.21	1.21	1.58
Zhejiang	1.67	1.80	2.64	2.52	2.74	2.80	2.64	1.76	2.59
Fujian	1.30	1.31	1.58	1.20	1.87	1.21	1.07	1.58	2.12
Guangxi	0.96	0.81	0.10	1.01	0.98	0.62	0.25	1.30	1.21
Guizhou	0.92	1.29	1.16	2.13	2.47	2.13	1.73	2.51	1.43
Sichuan	0.53	0.06	0.12	0.09	0.27	0.22	0.62	0.18	0.26
Guangdong	0.33	0.80	0.29	0.63	0.57	0.73	0.49	0.09	0.00
Jiangsu	0.14	0.01	0.18	0.01	0.00	0.15	0.02	0.00	0.03
Shanghai	0.07	0.00	0.23	0.14	0.00	0.05	0.01	0.01	0.10
Shaanxi	0.05	0.00	0.00	0.00	0.04	0.05	0.00	0.01	0.00
Henan	0.01	0.01	0.26	0.00	0.09	0.01	0.14	0.17	0.24
Total	26.98	25.38	27.04	30.26	28.49	33.55	28.47	26.05	28.77

The distribution pattern of *C. henryi* under the current climate was simulated by MaxEnt. The results were in high agreement with the actual occurrence record (Figures 1 and 5). The excellent habitats (>0.75) were mainly located in southwestern Zhejiang, northern Fujian, Jiangxi, southwestern Anhui, southeastern Hubei, southern and northwestern Hunan, northwestern Chongqing, southeastern Guizhou, and central Guangxi. Some excellent habitats occurred sporadically in Shanghai, Jiangsu, and Guangdong. Good habitats (0.50–0.75) were mainly found in northern Zhejiang, southern and western Anhui, southeastern Fujian, central Hubei, central‐northern Hunan, northern Guangdong, northern Guangxi, Guizhou, and parts of Sichuan. The remaining habitats were fair (0.25–0.50) and poor (<0.25), distributed in most parts of China.

The main excellent habitats of *C. henryi* occurred south of the Yangtze River basin and north of the Nanling Mountains, with the provinces of Zhejiang, Jiangxi, Anhui, Hubei, Hunan, Chongqing and Guizhou forming the core range. Extending away from the excellent core toward the north and south, habitat suitability decreased gradually to good, fair, and poor (Figure 5). In addition, the simulation results displayed that Taiwan and Yunnan, despite their subtropical conditions, were not suitable for *C. henryi* under the current climate scenario.

### 3.4 Changes in distribution under different climate scenarios

**FIGURE 6:**
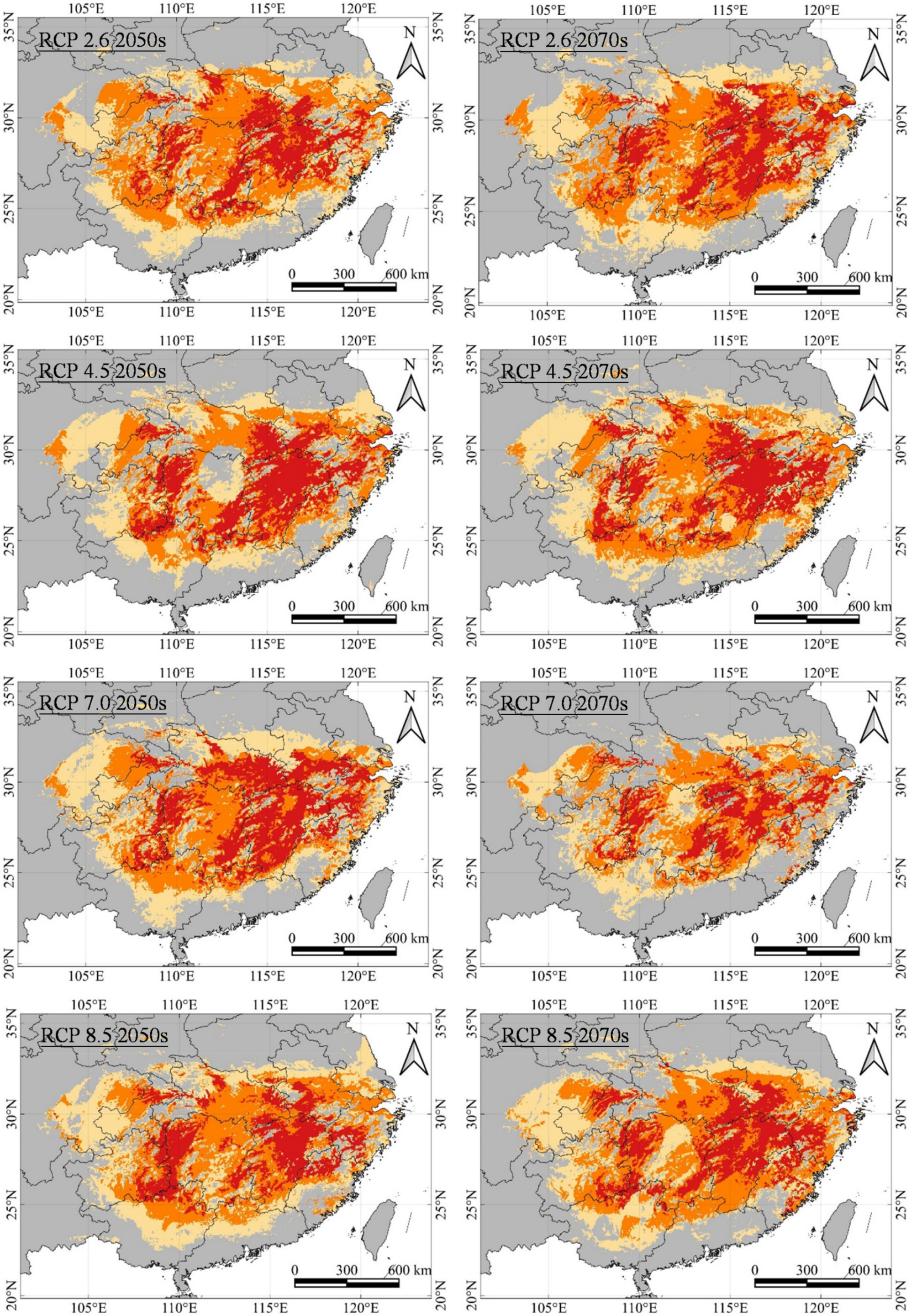
MaxEnt modeling of *C. henryi* based on four future climate‐change scenarios in 2050s and 2070s at RCP2.6, RCP4.5, RCP7.0, and RCP8.5. The habitat suitability categories include poor (0–0.25), fair (0.25–0.50), good (0.50–0.75), and excellent (>0.75), denoted by gray, light yellow, orange, and red, respectively.

Compared with the potential distribution area under the current climate scenario, suitable habitats for *C. henryi* would change under four future climate scenarios in the 2050s and 2070s (Table 4; Figure 6). Under future climate scenarios, the potential distribution area of the poor tier increases slightly by 1%–2%. In the fair tier, the potential distribution area of each climate scenario reduces notably compared with the current pattern, such as 5.59% (51.32 × 10^4^ km^2^) at RCP8.5–2050s and 18.83% (44.29 × 10^4^ km^2^) at RCP7.0–2070s. In the good tier, the potential distribution area decreased by 0.01% (53.34 × 10^4^ km^2^) in the RCP2.6–2050s and by 25.23% (39.89 × 10^4^ km^2^) in the RCP4.5–2050s. Interestingly, in the excellent tier, there is an increase in the potential distribution area with the largest expansion of 24.33% (33.55 × 10^4^ km^2^) in the RCP7.0–2050s, except for RCP2.6–2050s and RCP8.5–2050s.

Jiangxi Province would have the largest excellent‐habitat area for *C. henryi* in all future climate scenarios, bringing some fluctuations under the four scenarios in 2050s and 2070s (Table 5). Under the RCP8.5 (2050s) climate scenario, the excellent‐habitat area of *C. henryi* in Jiangxi would be 7.56 × 10^4^ km^2^, accounting for 29.02% of China. However, it would register a decrease of 20.84% compared with the current climate scenario. On the contrary, the excellent‐habitat area of Jiangxi under RCP4.5 (2050s) would be 10.41 × 10^4^ km^2^, increasing by 9.01% compared with the current climate. The trends in the excellent‐habitat area in other provinces would be similar to Jiangxi. Thus, medium emission intensities favor excellent‐habitat expansion, whereas excessively high emission intensities would shrink it.

## 4 DISCUSSION

### 4.1 Evaluating the MaxEnt model

In this study, the model successfully predicted the suitable habitats of *C. henryi* under different climate scenarios (Figure 2). From various MaxEnt modeling studies, two main generalizations could be distilled. First, the species' suitable habitats tended to move northwards (in the northern hemisphere) under the influence of future climate change (Akyol et al., 2020; Zhang, Zhu, et al., 2022). Second, different species were specific in their response to climate change, resulting in a spectrum of suitable habitat changes ranging from expansion to shrinkage compared with the current patterns (Gebrewahid et al., 2020; Pramanik et al., 2018). The findings could offer crucial information for the protection, management, and sustainable use of plant resources and address the challenges of global climate on important plant species.

The MaxEnt modeling technique has strong predictive power but has some limitations (Feng et al., 2019; Schnase et al., 2021). The model assumes a conservative ecological niche demand, but various factors such as environmental variables, occurrence records, and regional economics could exert joint influence on the prediction results.

More comprehensive species occurrence records can raise the modeling accuracy of species distribution (Feeley & Silman, 2011). Adequate and representative occurrence records constitute the basis for model construction and improve prediction accuracy (Hernandez et al., 2006). Our MaxEnt modeling yielded a mean AUC over 0.928 ± 0.014 (Figure 2), indicating good prediction results. It has been shown that integrating data from multiple sources could overcome the quality limitations of a single data source and improve prediction accuracy (Zhang et al., 2020). In addition, using multisource data could overcome the challenge of collecting species information in complex environments and broaden the application scope of ecological niche models (Chen, 2021). However, the method developed in our study focused on the influence of the spatial distance between samples, with less attention to sample size (referring to the 212 high‐quality and reliable distribution records of *C. henryi* selected by the sampling procedures explained in Section 2.1 Collecting species occurrence data) and spatial autocorrelation issues.

This study used environmental data from WorldClim, which were an average of 1970–2000 (Afrianto, 2021). Species distribution patterns have changed significantly in the last 20 years in response to intensifying climate change (Warren et al., 2018). Lacking 20 years of recent climate data may compromise the accuracy of prediction results, which could deviate from the actual situation. Moreover, some discrepancies between the data obtained by interpolation and the actual one are unavoidable (Pellicone et al., 2018). Therefore, we reckoned that the predicted suitable habitats of *C. henryi* could be broader than the actual coverage.

This study used nine bioclimatic variables to predict the suitable habitats of *C. henryi*. Other natural and cultural factors such as soil, topography, and land use could influence species distribution (Chauvier et al., 2021). Moreover, the factors could interact to generate synergistic or antagonistic effects. Future studies could improve the prediction power of modeling by evaluating the combined effects of various factors. In simulating species' potential range changes and future suitable habitats, more SDMs could be enlisted to enhance the study scope and accuracy (Duan et al., 2014; Rosner‐Katz et al., 2020).

### 4.2 Current distribution patterns and limiting factors

Hydrothermal conditions (climate) governed the geographical distribution of vegetation (Hessburg et al., 2019), with thermal conditions influencing the latitudinal change from south to north (in the northern hemisphere) and moisture conditions influencing the regional change from coastal to inland (Noor et al., 2020). Our modeling results identified Bio2, Bio11, and Bio15 as the key enabling factors of *C. henryi* distribution (Table 2; Figure 3). Studies on the geographical distribution of deciduous species of the Fagaceae family, such as deciduous oaks (*Quercus* spp.), related to and sharing similar botanical and ecological traits as *C. henryi*, may throw light on current and future distribution. The impact of low temperature in winter can influence the large‐scale range change of deciduous oak, with the cold index limiting the northward spread (Tian, 2007). The mean temperature of the driest quarter (winter in the study region) was likely the main factor restricting, for instance, *Q. chenii* from growing in the north (Li et al., 2016). Among the thermal variables, growing season warmth was most important for Beech (*Fagus* spp.) distribution. However, low winter temperature (coldness and mean temperature for the coldest month) and climatic continentality also influenced Beech occurrence (Fang & Lechowicz, 2006). This study did not find suitable habitats for *C. henryi* in northern China, probably because of the low winter temperature. In addition, *C. henryi* had a more confined northward spread than *Q. chenii*, indicating a lower tolerance to low temperature. Thus, temperature presents a critical environmental factor restricting the northward spread of *C. henryi*.

The temperature significantly influenced the seed germination for *C. henryi*. The optimum temperature for *C. henryi* seed germination is 25°C, below or above which the germination rate and growth of cone chestnut would be inhibited. Meanwhile, seeds could not germinate at 0°C (Li et al., 2020). This temperature control of the germinal stage may constitute a principal limiting factor of its spread. The temperature constraint on the distribution of several Fagaceae species has been demonstrated (Çoban et al., 2020; Conedera et al., 2004; Deng et al., 2018; Fang & Lechowicz, 2006; Li et al., 2016; G. Liu & Fang, 2000; Tulowiecki, 2020; Zhang, Zhu, et al., 2022). Figures 5 and 6 show a tendency of *C. henryi* to move its suitable habitats toward the eastern and northern regions of China, a process that could be driven by global warming.

However, excessively high or low winter temperatures can also limit the flowering of *C. henryi* (Ouyang, 2017). This phenomenon could be related to the annual temperature amplitude as one of the most critical factors of plant growth, development, and flowering. Most plants have to go through the annual cycle of warm‐cold‐warm phases, requiring the chilling or vernalization treatment for proper phenological expressions (Khodorova & Boitel‐Conti, 2013).

Moisture can influence *C. henryi* distribution with a weaker effect than temperature. Due to the different impacts of the southeast Pacific monsoon and the southwest Indian Ocean monsoon, China's subtropical region is divided into two subregions: the humid east with an average annual precipitation of 1000–2000 mm and the semihumid west of 900–1200 mm (Zhang et al., 2018). The precipitation conditions in the distribution areas of *C. henryi* under the current climate scenario aligned with the regional rainfall pattern (Table 3). Changes in the geographical distribution of vegetation from the coastal to inland latitudes were often governed by a corresponding moisture gradient. Seed germination and plant growth are directly influenced by water availability (Li, Li, & Fang, 2018; Liu, Zhao, et al., 2021). Abundant precipitation in winter and spring is a prerequisite for preserving or surviving Chinese endemic plants in East and Central China (Huang et al., 2015). The seeds of most plants mature in the fall. However, dehydration‐sensitive seeds tend to die if the winter is too dry (Nepomuceno, 1998). Due to the delayed response of plant physiology to precipitation factors, winter precipitation can exercise control on the dormancy and flowering period of subtropical tree species (Xiao et al., 2021).

In summary, climatic factors can further regulate the distribution pattern of *C. henryi* by controlling its growth and developmental rhythms (Figure 4). We found that Bio11 restricted the northern spread of *C. henryi* at the northern boundary of the subtropics (Qinling‐Huaihe), Bio10 limited the southern spread toward the tropics, and Bio15 controlled the westward spread. The combined effect of these bioclimatic factors was the range circumscription of *C. henryi* to subtropical China.

### 4.3 Differential range changes under future climate scenarios

Under the RCP7.0 scenario (2050s), the excellent habitats of *C. henryi* occupied the largest area, indicating that moderate climate change could expand the range (Table 5). Similar to other climate scenarios, its potential suitable habitats would concentrate in Jiangxi, Anhui, and western Hunan. Meanwhile, the excellent habitats in central Guangxi would disappear in the future and tend to migrate northward (Figure 6).

In response to future climate change, the distribution area of some species may increase, such as *Pinus pinea* (Akyol et al., 2020), *O. abyssinica* (Gebrewahid et al., 2020), and *Cyclobalanopsis glauca* (Zhang, Zhu, et al., 2022), including *C. henryi*. However, other species' ranges would contract due to global warming (Abdelaal et al., 2019; Abolmaali et al., 2018; Abrha et al., 2018). Why would the suitable habitats of *C. henryi* increase under climate change? *C. henryi* has an inherently wide horizontal and vertical distribution range, reflecting a certain tolerance of environmental stresses to facilitate range expansion. In addition, the ecological habits of *C. henryi* with a marked preference for warm and humid conditions could take advantage of the increasing favorable setting brought by climate change. Warmth‐preferring species, including *C. henryi*, would therefore continue to benefit from moderate climate change (Liu, Li, et al., 2021).

Under future climate change, the total suitable area of *C. henryi* would increase and expand northward (Figure 6). This is mainly represented by the enlargement and northward shift of the excellent habitats, most of which occur north of the Nanling Mountains (Figure 6). However, the northward range shift would reduce excellent habitats in southern China (especially in Guangdong and Guangxi). Some underlying processes can explain the anticipated modifications in the spatial patterns. First, moderate climate change can usher the positive effect of range expansion for warmth‐preferring species. Second, for the northern hemisphere, future climate change may raise the precipitation intensity in the middle‐high latitudes and increase the drought duration in the middle‐low latitudes (Liu et al., 2019). The net result would be a northward shifting of the suitable habitats.

However, the excellent‐habitat area for *C. henryi* would decrease under the RCP8.5 future climate scenario, indicating that relatively extreme warming could shrink its range. In recent years in China, warming has led to a significant increase in the extreme high‐temperature index in various regions (Jiang et al., 2016) to trim the distribution of *C. henryi*. In addition, the summer precipitation has been projected to decrease significantly in Central and Western China by the end of the 21st century under a warming scenario, which would induce the loss of some excellent habitats of *C. henryi* (Zhang, Hu, et al., 2022).

Under various future climate scenarios, the excellent habitats of *C. henryi* would increase in the following ascending order: RCP7.0 (2050s) > RCP4.5 (2050s) > RCP8.5 (2070s) > RCP4.5 (2070s) > RCP7.0 (2070s) > RCP2.6 (2070s) > Current > RCP8.5 (2050s) > RCP2.6 (2050s) (Figure 6; Table 4). This result provides additional evidence that too high temperatures are not suitable for *C. henryi*, an observation consistent with previous analyses.

Compared with the current climate scenario, the excellent‐habitat area of *C. henryi* should increase under future climate scenarios. Nevertheless, the range extension is primarily limited to the central‐eastern part of China. The differential responses to climate change in different areas could be attributed to the influence of extraneous factors. Some biotic, abiotic, and human variables, including species invasion, seed predation, lacking dispersal agents, disease infestation, land degradation, and resource overexploitation, may act independently or in concert to mold species distribution (Naudiyal et al., 2021). For example, soil and land use data for various climate‐change scenarios are unavailable. Thus, their effects are unknown and not analyzed in the study. Such uncertainties on the adaptive distribution of *C. henryi* could be reduced if suitable soil and land use conditions are taken into account. We acknowledged that the bioclimate envelope technique could offer a useful first approximation of the potentially severe impact of climate change on species distribution, despite the complexity of the natural system placing basic restrictions on predictive modeling (Pearson & Dawson, 2003). Overall, the critical role of climate in regulating the geographical distribution of species and indirectly controlling other biotic and abiotic factors has been supported by ample evidence (Chakraborty et al., 2016).

Although the excellent‐habitat area of *C. henryi* increased under various climate scenarios in future, the model results also indicated that too high temperatures were unsuitable for its growth (Figure 6; Table 4). Even warmth‐preferring species may become extinct because the negative impacts of climate change may surpass any potential advantages, such as increased temperature or a minor increase in CO_2_ levels (Bussotti et al., 2014; Sarikaya et al., 2022). Therefore, we should be cautious about the impact of climate change on the species' geographical range. Forest and conservation departments should pay more attention to maintaining *C. henryi* populations in the excellent habitats according to the present findings.

### 4.4 Implications of germplasm resources on species conservation

Exploring species' responses to future climate change can grasp the ecological risks they face, which are scientifically significant in formulating conservation strategies. Under different climate scenarios, there should be potential for future expansion of the excellent habitats for *C. henryi*. However, extreme climate change that would put most species at greater risk should be addressed in advance. The crucial roles of germplasm resources in conserving and managing species vulnerable to climate‐change impacts could be subject to detailed investigations.

Global warming has increased the frequency and severity of pest and disease outbreaks (Langridge et al., 2021; Logan et al., 2003), which may lead to the emergence of Chestnut blight in *C. henryi* populations in China. The disease has devastating impacts on the species (Rigling & Prospero, 2018). It was first discovered in *Castanea americana* in New York, USA, in the early 20th century. In the ensuing decades, the disease spread aggressively to decimate the American Chestnut trees in North America (Rigling & Prospero, 2018; Schlarbaum et al., 1998). From the 1930s, the Chestnut blight spread over a large area in Europe, causing severe damage to *C. sativa* (Ahmad & Baric, 2022). As one of the three major *Castanea* species endemic to China, the economic and nutritional values of *C. henryi* are high. Chestnut blight could reduce the yield and kill many individuals, bringing substantial economic losses and serious ecological hazards (Ye et al., 2022). Therefore, strenuous efforts should be instituted to curb its outbreak, the infectivity of which could be aggravated by global warming. It is an urgent task to accelerate the selection and breeding of new varieties with strong resistance to the disease (Jacobs, 2007).

We should strengthen the conservation of *C. henryi* germplasm resources and develop effective measures to protect it from the possible adverse effects of future climate change. First, further field research should be conducted on the community and population ecology of *C. henryi*. (Gong & Chen, 1997; Li et al., 2020, 2021; Li, Sun, et al., 2018; Liang et al., 2018; Ma et al., 2013; Wang et al., 2020; Yang et al., 2019). The dynamic monitoring of natural populations with fixed sample plots should be carried out to better understand environmental‐change impacts on *C. henryi* populations. Appropriate protection, development, and utilization measures should be formulated according to the monitoring results. The distribution pattern of *C. henryi* under different climate scenarios showed a higher concentration in Jiangxi, Anhui, Hunan, and Zhejiang, with more excellent habitats. Therefore, in situ conservation should be implemented in natural forests with a high concentration of *C. henryi* population, and germplasm resource protection and dynamic monitoring areas should be established. Second, plantation forest and edible cultivation of *C. henryi* should be enhanced by germplasm resource collection and selection, seedling cultivation, artificial mixed forest establishment and management, and large‐diameter timber tree cultivation. *C. henryi* should be introduced in predicted suitable areas and cultivated more widely in subtropical areas of China.

Based on the prediction results in relation to current and future climate, we propose classifying the excellent habitats of *C. henryi* into three types, each to receive tailor‐made treatments: preservation areas, loss areas, and new areas. First, the preservation areas may become a haven for *C. henryi* to cope with climate change under different greenhouse gas concentration gradients. The attention can therefore focus on protection and management. Second, the reasons for the losses of *C. henryi* should be analyzed. An in situ conservation model should be established. Under future climate scenarios, some South China and Southwest China areas would experience different degrees of loss. Such areas may no longer be suitable for *C. henryi* in the future. We suggest reinforcing studies on reproductive biology, population ecology, and artificial cultivation of *C. henryi* in the loss areas to avoid the risk of losing the genetic resources. Finally, the new area should be actively recruited as introduction spaces for *C. henryi*. Understanding the species' biological and ecological characteristics, appropriate locations could be identified to demonstrate introduction cultivation. A comprehensive spatial plan could be developed to realize extensive planting in suitable areas.

## 5 CONCLUSION

The suitable habitats of *C. henryi* in China were predicted by MaxEnt modeling under the current climate and four future climate‐change scenarios (RCP2.6, RCP4.5, RCP7.0, and RCP8.5), generating good results with a high degree of accuracy. The results of the study were not identical to our initial assumption, which supported the idea that the suitable habitat should shrink under RCP8.5. However, other future climate scenarios were inconsistent with the assumptions. Under the current climate scenario, the potential biogeographical range of the warmth‐preferring *C. henryi* is mainly found in the subtropical zone covering southern and central China provinces. The critical environmental factors regulating its growth are mean diurnal range (Bio2), mean temperature of the coldest quarter (Bio11), precipitation seasonality (Bio15), isothermality (Bio3), and annual precipitation (Bio12), with the optimal conditions at 8.21°C, 6.99°C, 60.37%, and 27.66%, respectively. Overall, the low temperature in winter is the most critical climate factor limiting the distribution of *C. henryi*. Under future climate‐change scenarios (except RCP2.6 and RCP8.5 in 2050s), the potential area of excellent habitats of *C. henryi* would increase significantly. At the same time, the excellent suitability habitats would migrate and expand northwards but shrink on the southern edge of South China. The findings may help timely and suitable responses to the effects of climate change on distribution, growth, research direction, economic use, management, and conservation.

## AUTHOR CONTRIBUTIONS

**Chunping Xie:** Data curation (lead); methodology (lead); software (lead); writing – original draft (lead). **Erlin Tian:** Data curation (equal). **Chi Yung Jim:** Formal analysis (lead); software (equal); visualization (equal); writing – review and editing (lead). **Dawei Liu:** Investigation (equal); supervision (equal); visualization (equal). **Zhaokai Hu:** Investigation (equal).

## CONFLICT OF INTEREST

The authors declare that they have no known competing financial interests or personal relationships that could have appeared to influence the work reported in this paper.

## Data Availability

The data that support the findings of this study are available online: 4TU Research Data. https://doi.org/10.4121/21257379. The data are not publicly available due to privacy or ethical restrictions.
